# Preparation of Aliphatic Hydroxamic Acid from *Litsea cubeba* Kernel Oil and Its Application to Flotation of Fe(III)-Activated Wolframite

**DOI:** 10.3390/molecules29010217

**Published:** 2023-12-30

**Authors:** Jingjing Xiao, Peiwang Li, Rukuan Liu, Qi Deng, Xudong Liu, Changzhu Li, Zhihong Xiao

**Affiliations:** 1 State Key Laboratory of Utilization of Woody Oil Resource, Hunan Academy of Forestry, Changsha 410004, China; xjj0806@126.com (J.X.); lindan523@163.com (P.L.); liurukuan@gmail.com (R.L.); dengqibq@163.com (Q.D.); lxd1995621@163.com (X.L.); 2 Key Laboratory of State Forestry and Grassland Administration on Utilization Science for Southern Woody Oil Resource, Hunan Academy of Forestry, Changsha 410004, China; 3 Hunan Provincial Key Laboratory of Oils and Fats Molecular Structure and Function, Changsha 410004, China

**Keywords:** *Litsea cubeba* kernel oil, aliphatic hydroxamic acids, Fe(III)-activated wolframite, flotation, hydrophobic

## Abstract

*Litsea cubeba* is a characteristic woody oil resource in Hunan. As a solid waste of woody oil resources, *Litsea cubeba* kernels are rich in *Litsea cubeba* kernel oil with a carbon chain length of C10–12 fatty acid. In this work, aliphatic hydroxamic acids (AHAs) with carbon chain lengths of C10–12 were prepared from *Litsea cubeba* kernel oil via methylation and hydroximation reactions. The adsorption and hydrophobicity mechanism of AHA towards wolframite was explored by contact angle, zeta potential, Fourier transform infrared spectroscopy (FTIR) and X-ray photoelectron spectroscopy (XPS). The flotation results demonstrated that AHA was a superior collector than the traditional collector such as benzoyl hydroxamic acid (BHA). Zeta potential and contact angle results have shown that AHA was adsorbed on the surface of the Fe(III)-activated wolframite in its anionic form, which significantly improved the surface hydrophobicity of wolframite. FTIR and XPS revealed that AHA was chemically adsorbed on the surface of Fe(III)-activated wolframite in the form of a five-member ring, which made the hydrophobic chain reach into the solution, come in contact with bubbles, and achieve flotation separation.

## 1. Introduction

As an important strategic metal, tungsten is widely used in military applications, aerospace, light industry, textiles, electronic technology, and the chemical industry [1,2,3]. Wolframite ((Fe,Mn)WO_4_) is a very important mineral raw material used for tungsten refining. Flotation is one of the most important means to obtain high-purity wolframite. The collector is a key factor and core technology used to realize the separation and enrichment of wolframite via foam flotation. Hydroxamic acid, which contains the -C(=O)NHOH functional group in its molecular structure, can form a ring with a metal atom, so it is often used as a flotation collector for tungsten ore [4,5].

At present, the main wolframite collectors used for industrial applications are benzohydroxamic acid, salicylhydroxamic acid, C5–9 hydroxamic acid, and naphthohydroxamic acid [6,7]. However, these traditional hydroxamic acids have some problems in practical applications, such as weak collecting ability, large pharmaceutical consumption, and low flotation recovery rate of tungsten ore [8,9]. This is mainly due to the short carbon chain of the hydrophobic groups in traditional hydroxamic acids and weak molecular hydrophobic ability. Therefore, metal ions must be added as activators in the flotation process to promote the interaction between hydroxamic acid and wolframite mineral in order to obtain an ideal recovery rate of wolframite [10,11,12]. Bivalent metal ions such as Pb^2+^, Mn^2+^, and Fe^2+^ have been widely used in the activated flotation separation of wolframite as activators [2,4,13], but the active flotation of trivalent metal ion such as Fe^3+^ in wolframite has been rarely reported.

After essential oil extraction, ~200,000 tons of solid waste of *Litsea cubeba* kernels are obtained every year. According to the literature, the solid waste obtained from *Litsea cubeba* kernels contains ~25% of *Litsea cubeba* kernel oil, which mainly contains 10 and 12 carbon fatty acids. Furthermore, hydroxamic acid with medium and long carbon chains (8–12 carbons) has been widely used in mineral flotation, corrosion inhibition, and other chemical industries [14,15,16]. Therefore, *Litsea cubeba* kernel oil is a suitable raw material for the preparation of hydroxamic acid, which also provides a new way to utilize *Litsea cubeba* kernel solid waste.

In this paper, the remaining solid waste obtained from the industrial extraction of *Litsea cubeba* essential oil was fully utilized and C10-12-containing aliphatic mixed hydroxamic acids (AHAs) were obtained from *Litsea cubeba* kernel oil via methylation and hydroximation, which was used as a flotation collector for wolframite before and after Fe^3+^ activation. Zeta potential and contact angle measurements were used to analyze the active form and hydrophobicity of the AHA on the wolframite surface before and after Fe^3+^ activation. Fourier transform infrared spectroscopy (FTIR) and X-ray photoelectron spectroscopy (XPS) were used to reveal the adsorption mechanism of the AHA on the wolframite surface after Fe^3+^ activation.

## 2. Results and Discussion

### 2.1. Preparation and Characterization of AHA

#### 2.1.1. Preparation of AHA

**Figure 1:**
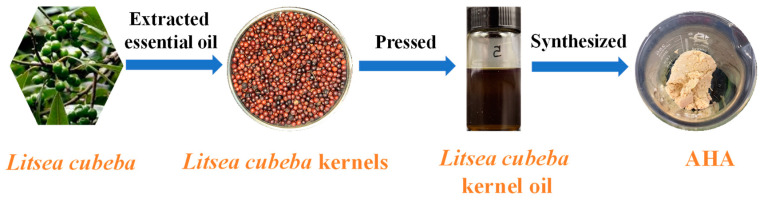
The preparation process of AHA.

After pressing, *Litsea cubeba* kernel oil was obtained from *Litsea cubeba* kernels. Then, the AHAs were prepared from *Litsea cubeba* kernel oil through methylation and hydroximation. The detailed preparation process is shown in Figure 1.

According to our analysis using gas chromatography, the main fatty acid composition of *Litsea cubeba* kernel oil is shown in Table 1.

A total of 20 g of *Litsea cubeba* kernel oil, 0.2 g of sulfuric acid, and 6 g of methanol were successively added into a 250 mL three-necked flask and heated at 60 °C for 90 min. After the reaction was complete, the fatty acid methyl ester product was obtained upon repeated washing with water three times. A total of 7.03 g of hydroxylamine hydrochloride, 8.09 g of sodium hydroxide, 40 mL of water, and 20 mL of methanol were successively added into a 250 mL three-necked flask. At 10 °C, the above crude fatty acid methyl ester products were added dropwise into the three-necked flask. The temperature was increased to 50 °C for 4 h and 37.55 g of the AHA products were obtained. The reaction is shown in Equation (1).

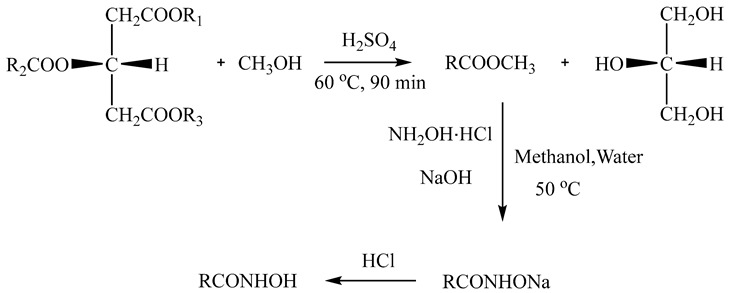
(1)


#### 2.1.2. Characterization of AHA

The synthesized n-hydroxy-decamides, n-hydroxy-dodecamides, and AHA were characterized using nuclear magnetic and infrared spectroscopy to determine the target products.

N-hydroxy-decamides: White flake crystal. ^1^H NMR (DMSO-*d*_6_, 500 MHz), δ: 10.31 (s, 1H, OH), 8.64 (s, 1H, NH), 1.91–1.95 (t, 2H, -CH_2_-), 1.46–1.49 (t, 2H, -CH_2_-), 1.24 (s, 12H, -CH_2_-), 0.85–0.88 (t, 3H, -CH_3_). FTIR (KBr): 3246 (N-H), 3061 (O-H), 2916–2842 (C-H), and 1662 (C=O) cm^−1^.

N-hydroxy-dodecamides: White flake crystal. ^1^H NMR (DMSO-*d*_6_, 500 MHz), δ: 10.33 (s, 1H, OH), 8.66 (s, 1H, NH), 1.91–1.95 (t, 2H, -CH_2_-), 1.45–1.49 (t, 2H, -CH_2_-), 1.24 (s, 16H, -CH_2_-), 0.84–0.87 (t, 3H, -CH_3_). FTIR (KBr): 3251 (N-H), 3066 (O-H), 2910–2842 (C-H), 1662 (C=O) cm^−1^.

**Figure 2:**
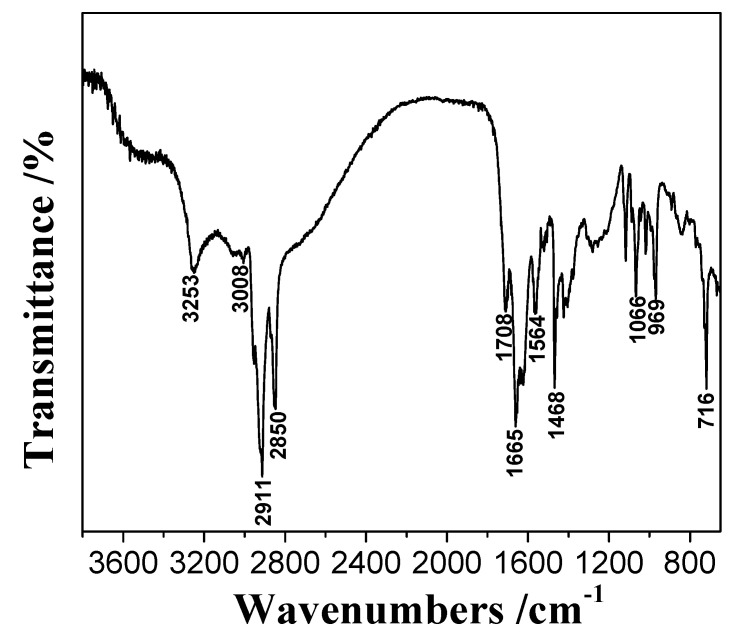
FTIR spectroscopy of AHA.

AHA: Off-white solid. FTIR (KBr disks, see Figure 2): 3253 (N-H), 3008 (O-H), 2911–2850 (C-H), 1665 (C=O) cm^−1^. The above analysis showed that the target product had been synthesized.

### 2.2. Micro-Flotation

**Figure 3:**
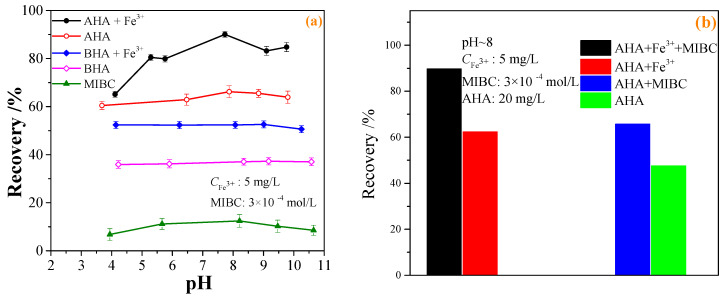

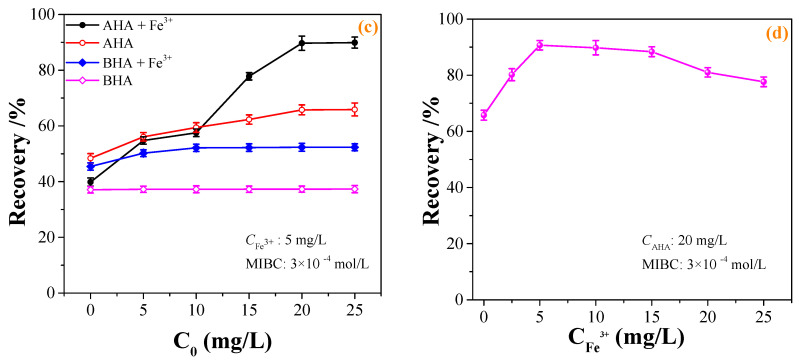
Effect of BHA and AHA as flotation collectors on wolframite flotation ((**a**) effect of pH; (**b**) effect of frother; (**c**) effect of collector concentration; (**d**) effect of iron ions concentration).

The effect of the pulp pH on the flotation recovery of wolframite performed at room temperature in the presence and absence of 20 mg·L^−1^ BHA and AHA collectors, and the addition of 5 mg·L^−1^ Fe^3+^ was shown in Figure 3a. Figure 3a showed that with an increase in the pH, the flotation recovery of wolframite by BHA and AHA first increased and then decreased. When compared with BHA, AHA had a stronger collecting capacity for wolframite. After adding Fe^3+^, the flotation recovery of wolframite by BHA increased from ~38% to ~52%. In the pH range of 5–9, the flotation recovery of wolframite by the AHA increased from ~60% to >80%. When the pH was ~8, the flotation recovery of wolframite by the AHA reached the highest value of ~90%. When compared with BHA, Fe^3+^ significantly promoted the flotation recovery of wolframite using AHA as a collector under weak acid to weak alkaline conditions. Figure 3a also showed that when only a frother was added without a collector, the flotation recovery of wolframite was no more than 15%. 

The effect of the frother on the flotation recovery of wolframite performed at room temperature, pH~8, an AHA concentration of 20 mg·L^−1^, and the addition of 5 mg·L^−1^ Fe^3+^ is shown in Figure 3b. Figure 3b indicated that in the absence of frother, the flotation recovery of wolframite was only ~48% when using AHA as collector, after the addition of Fe^3+^, the flotation recovery of wolframite achieved 66%. In the presence of the frother, the flotation recovery of wolframite could reach ~62% when using AHA as a collector; after being activated by Fe^3+^, the flotation recovery of wolframite increased to 90%. The results showed that the flotation recovery of wolframite can be significantly increased with the addition of frother.

The effects of the concentration of benzohydroxamic acid (BHA) and aliphatic hydroxamic acid (AHA) on the flotation recovery of wolframite at room temperature, pH ~8 and Fe^3+^ dosage of 5 mg·L^−1^ are shown in Figure 3c. Figure 3c displays that the flotation recovery of wolframite increased with an increase in the collector concentration. After adding Fe^3+^, the flotation recovery of wolframite increased from ~38% to ~50%, and the concentration change had little effect on the flotation of wolframite using BHA as the collector. The addition of Fe^3+^ had a significant effect on the flotation recovery of wolframite using AHA as the collector. When the AHA concentration was >20 mg·L^−1^, the flotation recovery of wolframite exhibited little change.

The effect of the Fe^3+^ concentration on the flotation recovery of wolframite performed at room temperature, pH ~8 and AHA concentration of 20 mg·L^−1^ was shown in Figure 3d. Figure 3d demonstrated that with an increase in the Fe^3+^ concentration, the flotation recovery of wolframite decreased slowly. When the concentration of Fe^3+^ was between 0 and 15 mg·L^−1^, the addition of Fe^3+^ promoted the flotation recovery of wolframite, but when the addition of Fe^3+^ was <2.5 or >15 mg·L^−1^, Fe^3+^ gradually inhibited the flotation recovery of wolframite. The results showed that only a small amount of Fe^3+^ needs to be added to significantly promote the flotation recovery of wolframite when using AHA as a collector.

### 2.3. Zeta Potential

**Figure 4:**
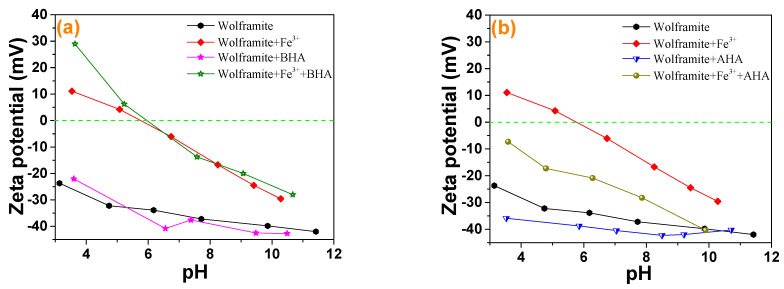
Zeta potential analysis of wolframite and Fe^3+^ treated wolframite before and after BHA (**a**) and AHA (**b**) treatment.

**Figure 5:**
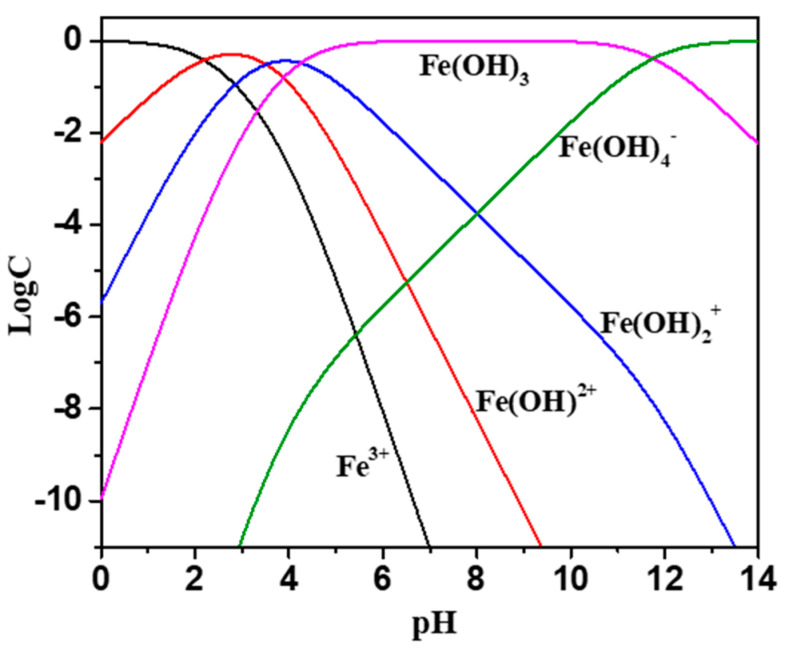
Distribution diagrams of 5 mg/L Fe^3+^ solution as a function of pH.

The effects of the collector and the addition of Fe^3+^ on the zeta potential of wolframite under different pH conditions were shown in Figure 4. There was no isoelectric point in wolframite in the pH range of 3–12 [17,18]. The presence of ionic species at different pHs with 5 mg·L^−1^ Fe^3+^ ions is presented in Figure 5. According to Figure 5, when pH > 6, the main species of Fe^3+^ ions is Fe(OH)_3_ precipitate, accompanied by a portion of Fe(OH)_4_^−^, Fe(OH)_2_^+^ and Fe(OH)^2+^ species. It was reported that the IEP of Fe(OH)_3_ precipitate is around pH 8 [19], which means that the positive charge around pH 8 is caused by Fe(OH)_4_^−^, Fe(OH)_2_^+^ and Fe(OH)^2+^ species. It can be deduced that the deviation of potential towards positive direction after the addition of Fe^3+^ is mainly attributed to the charge neutralization effect between negatively charged wolframite surface and iron hydroxide complexes. When Fe^3+^ was added, the zeta potential of wolframite increased and the negatively charged collector ions were more likely to be absorbed on the surface of Fe^3+^ activated wolframite. Figure 4a showed that the addition of BHA in pulp had little effect on the zeta potential of the wolframite surface before and after Fe^3+^ activation. Figure 4b indicated that the zeta potential on the wolframite surface prior to Fe^3+^ activation led to a small change after the AHAs were added to the pulp, and after Fe^3+^ activation, the zeta potential on the wolframite surface significantly decreased after the AHAs were added to the pulp, indicating that the AHAs were absorbed on the wolframite surface in the form of an anion. This phenomenon is consistent with the collecting ability of the different hydroxamic acids in our micro-flotation experiments.

### 2.4. Contact Angle

**Figure 6:**
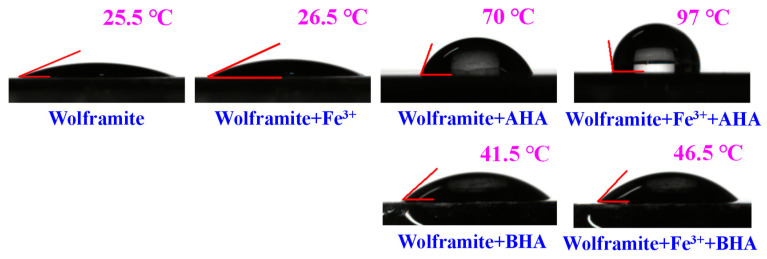
Contact angle analysis of wolframite and Fe^3+^ treated wolframite before and after BHA and AHA treatment.

The contact angle changes of the wolframite surface before and after Fe^3+^ activation and the action of the collector were shown in Figure 6. Figure 6 shows that the contact angle of the water droplets on the wolframite surface had little change before and after the addition of Fe^3+^, as expected. This is because inorganic metal cations (and their hydroxide precipitates) are hydrolyzed, and hydrophilic. Prior to Fe^3+^ activation, the contact angle of the water droplets on the wolframite surface after the adsorption by BHA for 2 h increased to 41.5° and the contact angle of the water droplets on the wolframite surface after adsorption by AHA increased to 70°. The contact angle of the water droplets on the wolframite surface after treatment with the AHA was much larger than that on the wolframite surface after treatment with BHA. This showed that, when compared with BHA, the surface hydrophobicity of wolframite treated using AHA was significantly enhanced.

After the Fe^3+^ activation, the contact angle of the droplet on the wolframite surface after adsorption by BHA increased to 46.5° and the contact angle of the droplet on the wolframite surface after adsorption by AHA increased to 97°, indicating that Fe^3+^ activation had little effect on the contact angle of the droplet before and after the action of BHA. The activation energy of Fe^3+^ significantly increased the contact angle of the wolframite surface.

### 2.5. FTIR Spectra Analysis

**Figure 7:**
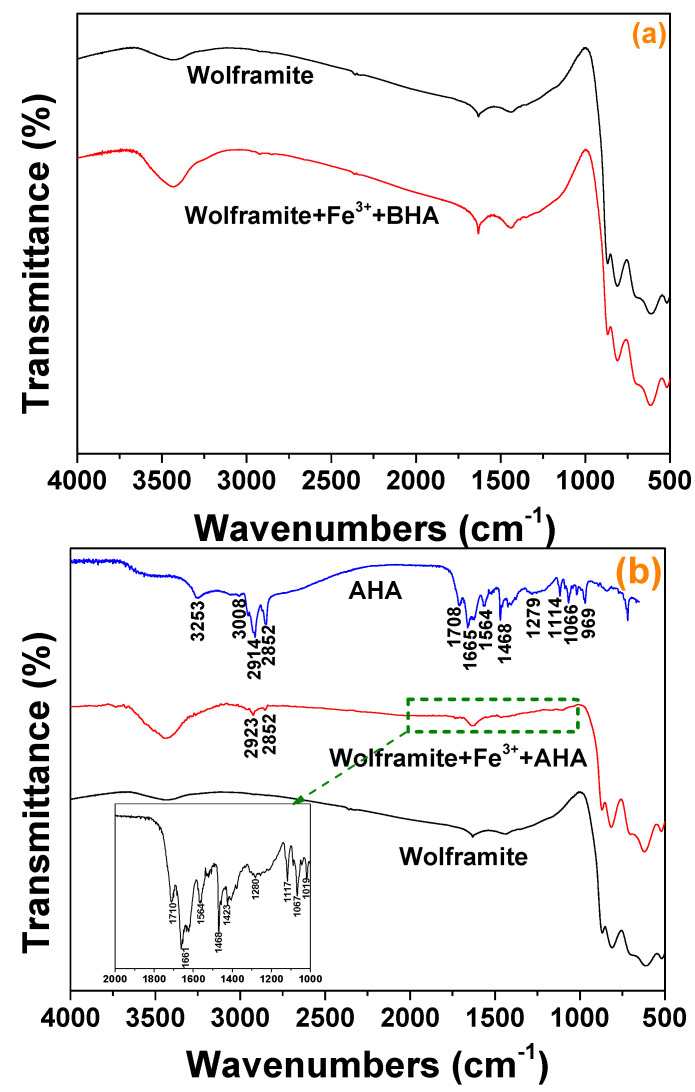
FTIR spectra of Fe^3+^ treated wolframite before and after BHA (**a**) and AHA (**b**) treatment.

The infrared spectra of hydroxamic acid before and after interaction with Fe^3+^ activated wolframite was shown in Figure 7. Figure 7a showed that the infrared spectrum obtained for the Fe^3+^-activated wolframite surface did not change significantly after BHA adsorption. Figure 7b indicated that after adsorption by AHA, the infrared spectrum of Fe^3+^-activated wolframite changed obviously, and the N-H and O-H stretching vibration peaks attributed to AHA at 3253 and 3008 cm^−1^ disappeared [16]. The stretching vibration peak attributed to methyl and methylene in the range of 2800–2900 cm^−1^ appeared on the surface of the Fe^3+^ activated wolframite and the characteristic absorption peak attributed to the AHA also appeared in the range of 1000–1700 cm^−1^. The results indicated that the AHA may be adsorbed on the surface of Fe^3+^ activated wolframite via chemisorption.

### 2.6. XPS Analysis

**Figure 8:**
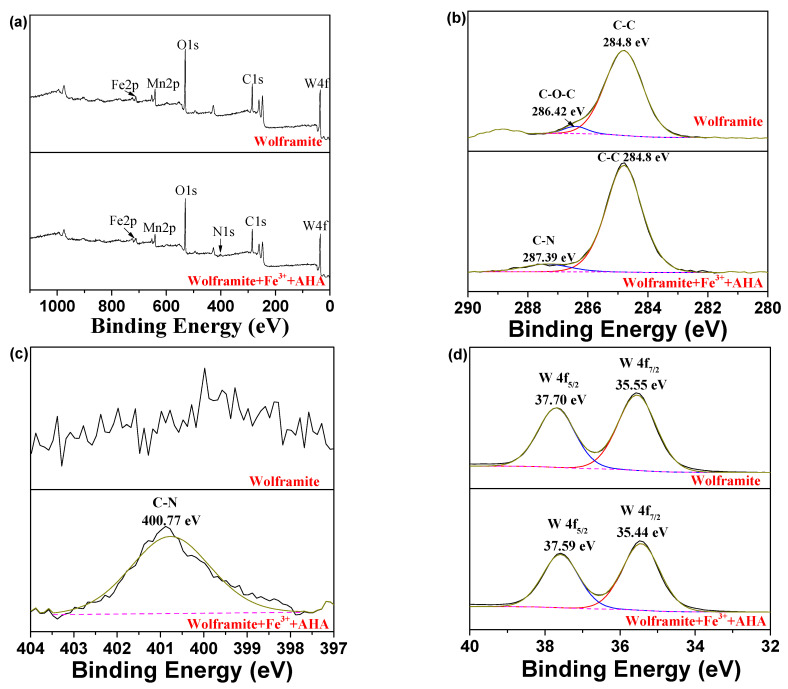

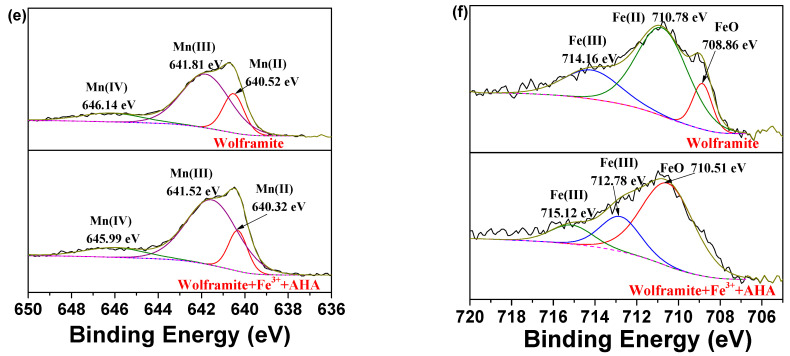
XPS spectra of wolframite and Fe^3+^ treated wolframite before and after AHA treatment ((**a**) survey; (**b**) C 1s; (**c**) N 1s; (**d**) W 4f; (**e**) Mn 2p; (**f**) Fe 2p).

The XPS spectra before and after the interaction of AHA with Fe^3+^ activated wolframite was shown in Figure 8. A new characteristic peak corresponding to N 1s appeared in the XPS survey spectrum of wolframite after adsorption by the AHA (Figure 8a), which confirmed the chemical adsorption of AHA on the wolframite surface, which was consistent with the infrared spectroscopy results.

Figure 8b displayed that two C 1s peaks were observed on the wolframite surface at 284.8 and 286.42 eV, which was attributed to the C-C and C-O-C bonds [20], respectively. These characteristic absorption peaks may be caused by external contamination of the test sample. When the Fe^3+^-activated wolframite was treated with the AHA, a new peak with a binding energy of 287.39 eV ascribed to the C-N bond appeared on the surface of wolframite. It can be inferred from this that the AHA were adsorbed on the surface of the Fe^3+^-activated wolframite via chemical action to form C-N bonds.

Figure 8c demonstrates, before treatment, the main N 1s peak on the wolframite surface was not obvious and disordered. After Fe^3+^ activation and AHA adsorption, a characteristic peak ascribed to C-N appeared on the wolframite surface at 400.77 eV [21], which was caused by the adsorption of AHA on the wolframite surface.

Figure 8d shows the W 4f XPS peaks of wolframite appear at 35.55 and 37.70 eV, which could be attributed to the W 4f_7/2_ and W 4f_5/2_ of the WO_4_^2−^ group [17,22], respectively. After Fe^3+^ activation and AHA adsorption, the W 4f XPS peaks in wolframite appeared at 35.44 and 37.59 eV with little change. Small negative beam energy transfers may be caused by charging effects or errors in the XPS measurement process [20].

Figure 8e indicated that Mn 2p_3/2_ XPS peaks in wolframite were observed at 640.52, 641.81, and 646.14 eV, which were attributed to the Mn(II), Mn(III), and Mn(IV) species [17], respectively. After Fe^3+^ activation and AHA adsorption, the Mn 2p_3/2_ XPS peaks corresponding to the Mn(II), Mn(III), and Mn(IV) species in wolframite appeared at 640.32, 641.53, and 645.99 eV, respectively, and the binding energy shifted negatively by −0.20, −0.29, and −0.15eV, respectively.

Figure 8f showed the Fe 2p_3/2_ peaks in wolframite mainly occurred at 708.86, 710.78, and 714.16 eV, which could be attributed to the Fe(III), Fe(II), and FeO species [23], respectively. After Fe^3+^ activation and AHA adsorption, the Fe 2p_3/2_ peaks in wolframite appeared at 710.51, 712.78, and 715.12 eV, which were attributed to the Fe(III), Fe(III), and FeO species, respectively. The change in binding energy showed that the chemical environment of the Fe atoms on the wolframite surface AHA obviously changed, indicating that Fe was the active site for AHA interaction with wolframite and the addition of Fe^3+^ can increased the flotation ability of the AHA to wolframite, which is consistent with the results of our micro-flotation experiments.

### 2.7. DFT Calculation

**Figure 9:**
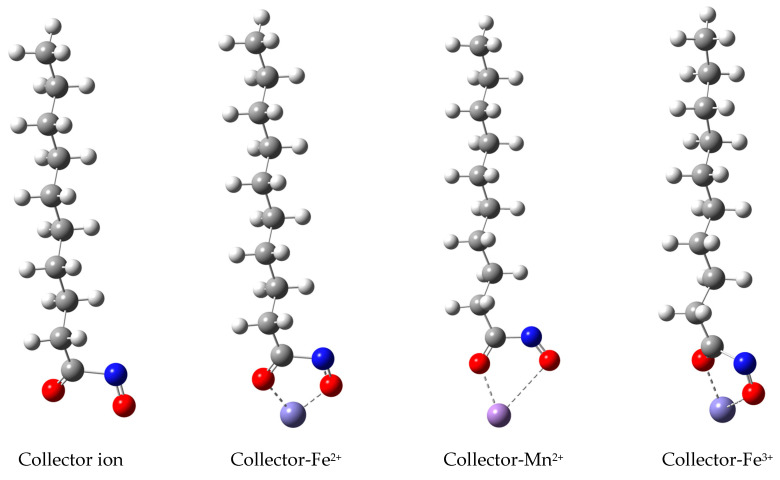
The binding model of N-hydroxy-decamide (red ball represent oxygen atom, blue ball represent nitrogen atom) with metal ions (Fe^2+^, Mn^2+^ and Fe^3+^).

The interaction energy between hydroxamic acid and metal ion was calculated by density functional theory. Taking N-hydroxy-decamides as an example, the geometric optimization structure of N-hydroxy-decamides ion and their corresponding metal complexes were listed in Figure 9, and the simulation results of the interaction between N-hydroxy-decamides and metal ions are shown in Table 2.

The binding ability of collectors to the metal ions, i.e., Fe^3+^, Mn^2+^ or Fe^2+^, would be relevant to the collecting ability. As shown in Table 2, The binding energy of the collector with Fe^3+^ is more negative than that with other metal ions (Fe^2+^ and Mn^2+^), indicating that the addition of Fe^3+^ can enhance the collection ability of the collector, which is consistent with the results of flotation experiments.

### 2.8. Discussion

The zeta potential and micro-flotation results showed that the adsorption/precipitation of Fe^3+^ on the negatively charged wolframite surface to make the zeta potential of wolframite shift to positive direction, then AHA absorbed on the surface of the Fe(III)-activated wolframite in their anionic form and exhibited a superior collecting ability to Fe(III)-activated wolframite. The contact angle results indicated that compared with BHA, AHA can significantly increase the hydrophobicity of wolframite surface before and after Fe^3+^ ions activation. This may be due to the hydrophobic chains of the AHA being long carbon chains (>9 carbons), while the hydrophobic chain of BHA was a benzene ring, and the hydrophobicity of the long carbon chains were stronger than that of the benzene ring.

Infrared analysis and XPS pointed out that the self-assembly and accumulation of AHA on Fe(III)-activated wolframite rendered the formation of surface AHA-Fe species where the five-member ring configurations (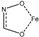) were generated. DFT calculations further elucidated that AHA and Fe^3+^ ions can form a more stable chelate.

**Figure 10:**
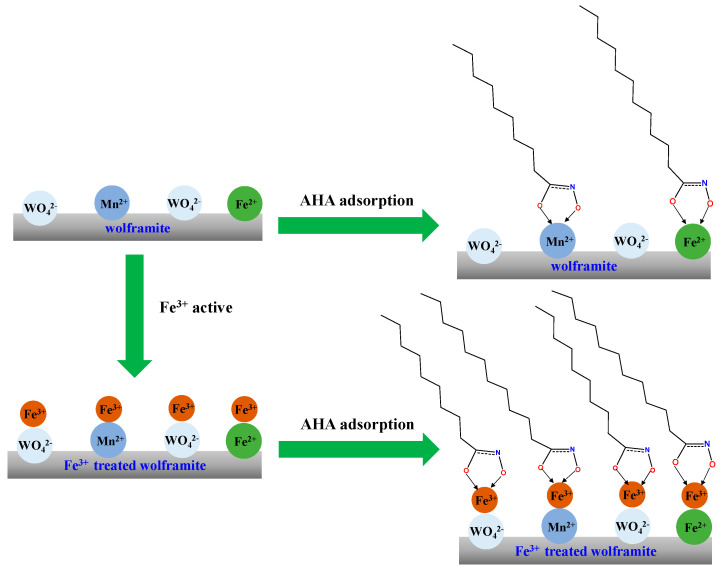
The suggested adsorption model of Fe^3+^ treated wolframite surface after AHA treatment.

Based on the above analysis, we propose a simple adsorption model between Fe(III)-activated wolframite and the AHA, as shown in Figure 10. The results showed that the AHA interacted with the Fe^3+^ active sites adsorbed on the surface of wolframite via the hydroxamic acid group in the molecule, making the hydrophobic long carbon chain in the molecular structure direct toward the water, showing stronger hydrophobicity than the benzene ring. Therefore, the AHA had stronger hydrophobicity for Fe(III)-activated wolframite than BHA.

## 3. Experimental Section

### 3.1. Materials

**Figure 11:**
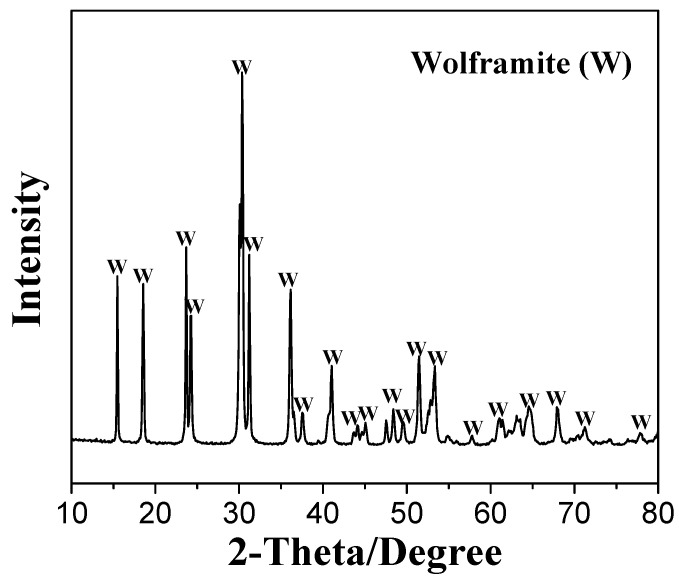
XRD spectrum of wolframite.

Wolframite ((Fe,Mn)WO_4_) samples were purchased from Guangzhou, Guangdong, China. The X-ray diffraction (XRD) and X-ray fluorescence (XRF) analyses of the wolframite samples are presented in Figure 11 and Table 3. After being crushed, hand-picked, and ground in an agate mortar, the bulk wolframite samples were made into particles with diameters of <76 μm. Samples with particle sizes of −76~+38 μm were used in the micro-flotation tests, while finer (<5 μm) particles were employed in the zeta potential, FTIR and XPS analyses.

AHAs were prepared in our laboratory, n-hydroxy-decamides and n-hydroxy-dodecamides also were synthesized according to the literature [24]. Benzohydroxamic acid (BHA) was purchased from Macklin Reagent Net with a purity of >98%. NH_2_OH·HCl, methanol (CH_4_O), ethanol (C_2_H_6_O), NaOH, KOH, KCl and methyl isobutyl carbinol (C_6_H_14_O) were all of the analytic grades and purchased from Macklin Reagent Net. Hydrochloric acid was purchased from Sinopharm Chemical Reagent Co., Ltd. (Shanghai, China) with a purity of 36–38%. Al_2_O_3_ powder was purchased from Shanghai Chenhua Instrument Co., Ltd. (Shanghai, China). All experiments were performed using deionized water if not specified otherwise.

### 3.2. Gas Chromatography

GC analysis was carried out as described in GB/T GB5009.168-2016 [25]. The fatty acids were qualitatively and quantitatively compared with a standard mixture of 37 kinds of fatty acids. The experiment was repeated three times and the average value was reported.

Gas phase conditions: Shimadzu GC-2010 Plus model gas chromatography analyzer (Shimadzu, Kyoto, Japan) with Flame Ionization Detector (FID). The capillary chromatographic column was Dura Bond HP-88 (column length 100 m, inner diameter 0.25 mm, film thickness 0.2 μm). Injector temperature: 250 °C; detector temperature: 280 °C. Temperature programming: initial temperature of 100 °C for 13 min; 100–180 °C, heating rate of 10 °C/min, maintained for 6 min; 180–200 °C, heating rate of 1 °C/min, keep 20 min; 200–230 °C, heating rate 4 °C/min, keep 10.5 min. A 25.0 mL/min high-purity helium as carrier gas; hydrogen flow rate: 30.0 mL/min; air flow rate: 300.0 mL/min; injection volume: 1.0 μL. The fatty acids were qualitatively and quantitatively compared with 37 fatty acid standard mixtures.

### 3.3. Micro-Flotation Tests

Micro-flotation experiments were carried out in a 220 mL Hallimond tube under a N_2_ atmosphere (200 ± 2 mL·min^−1^) for the whole flotation process, as reported in the literature [26]. 2 g of wolframite particles in an appropriate volume of distilled water were added to the Hallimond tube for each test, then dilute HCl and NaOH solutions were used to adjust the pH for 3 min. The Fe^3+^ ions were added and agitating took place for 3 min. The collector was introduced and stirring for 3 min, after the addition of a 3.0 × 10^−4^ mol·L^−1^ methyl isobutyl carbinol (MIBC) solution as a frother, the slurry was stirred for 1 min, and finally, the floated materials were collected for 4 min. After each flotation experiment, the foam products and tailings were dried and weighed, respectively. The recovery was calculated based on the dry weights of the products obtained. The calculated flotation recovery was averaged over three independent tests.

### 3.4. Zeta Potential Tests

The zeta potential was measured using a ZetaPlus zeta potential analyzer (Brookhaven Corporation, Holtsville, NY, USA). A total of 0.05 g of 5 µm wolframite particles was added to 50 mL of 1 × 10^−3^ mol·L^−1^ KCl solution in the presence and absence of 20 mg·L^−1^ AHA and BHA. After stirring for 5 min, the pH was adjusted using dilute KOH or HCl solution to the desired value. The agitated suspension was collected for the zeta potential measurements. The results presented are the average of three independent measurements with a typical variation of ±5 mV.

### 3.5. Contact Angle Measurements

The single mineral sample was embedded in epoxy resin, then hand-sanded in sequence with water on 80, 400, 800, and 1200 SiC sandpaper, and finally polished with 1 and 0.05 µm Al_2_O_3_ powder suspension on a polishing cloth. The polished ore sample was cleaned twice with ultra-pure water under ultrasonic wave, and then removed and dried using high-purity nitrogen. A JC2000C instrument (Shanghai Zhongchen Digital Technology Co., Ltd., Shanghai, China) was used to measure the contact angle measurement. Each measurement was repeated four times and the data reported as the average value.

### 3.6. FTIR Spectroscopy and XPS

FTIR spectroscopy and XPS of AHA, wolframite, and AHA-conditioned wolframite were recorded on a model 740 Fourier transform infrared spectrometer from Nicolet (Seoul, Republic of Korea) (via KBr disks) and Thermo Scientific ESCALAB 250Xi spectrometer (Thermo Fisher Scientific, Shanghai, China), respectively. The AHA-conditioned wolframite particles were prepared by agitating a suspension containing 0.5 g of wolframite and 50 mL of 20 mg·L^−1^ AHA solution for ~6 h at 298 K. After filtration and washing with distilled water, the AHA-conditioned wolframite samples were obtained and then stored in a vacuum oven.

The fitting and analysis approaches used for the XPS adsorption bands have been described in our previous study and the binding energies calibrated by setting the C 1s binding energy at 284.8 eV.

### 3.7. Theoretical Calculation Methods

All the quantum mechanical calculations were performed with Gaussian 09 software package [27]. Structural optimization was performed using the B3LYP functional (empirical dispersion correction GD3(BJ)). The basis set used for the C, H, O, and N atoms was 6-31G(d), whereas the SDD basis set was employed for the Fe and Mn atoms. The aqueous environment was simulated using the SMD model [28,29].

## 4. Conclusions

In this work, it is shown that the solid waste obtained from *Litsea cubeba* after the extraction of its essential oil has been fully utilized. The *Litsea cubeba* kernel oil was extracted by pressing *Litsea cubeba* kernel. After methylation and hydroximation, the target product AHA was obtained, which mainly contained C10-12 aliphatic hydroxamic acid. The flotation behavior and adsorption mechanism of wolframite before and after activation by Fe^3+^ using AHA as a collector have been studied by micro-flotation, zeta potential, contact angle, DFT calculation, infrared spectrum and XPS analyses. Based on the results of a series of experiments, the following conclusions were drawn.

Micro-flotation results demonstrated that AHA exhibited better flotation performance for wolframite than BHA under weak alkaline conditions and the addition of Fe^3+^ ions could be benefit to enhance the collecting ability of AHA for wolframite. The zeta potential and contact angle results showed that the AHAs were adsorbed on the active sites with positive charge on the surface of wolframite activated by Fe^3+^ ions, forming a hydrophobic film on the surface of wolframite, which increased the surface hydrophobicity and contact angle. FTIR and XPS results revealed that AHA self-assembled on Fe(III)-activated wolframite via forming surface AHA-Fe complexes where five-member ring configurations were formed. DFT calculations further demonstrated that hydroxamate groups were active parts in the reaction and AHA were more inclined to chelate with Fe^3+^ ions.

## Figures and Tables

**Table 1:** Fatty acid composition of *Litsea cubeba* kernel oil.

Serial Number	Retention Time /min	Retention Index	Component	Content	Name
1	21.174	1308	C10:0	16.433%	capric acid
2	24.593	1510	C12:0	66.893%	lauric acid
3	28.065	1710	C14:0	2.148%	myristic acid
4	31.506	1912	C16:0	1.095%	palmitic acid
5	35.665	2113	C18:0	0.561%	stearic acid
6	37.025	2084	C18:1	9.534%	oleic acid
7	39.408	2079	C18:2	2.995%	1inoleic acid
8	40.989	2309	C20:0	0.085%	arachidic acid
9	42.517	2072	C18:3	0.077%	1inolenic acid
10	42.703	2281	C20:1	0.179%	eicosenoic acid

**Table 2:** The calculated energies relevant to the chelation of collectors and metal ion species/a.u.

Complexes	E_C_	E_M_	E_S_	E_B_
Collector-Fe^2+^	−597.672161	−123.5570888	−721.27406	−0.0448102
Collector-Mn^2+^		−103.9976303	−701.794163	−0.1243717
Collector-Fe^3+^		−123.1967865	−721.071508	−0.2025605

Note: E_C_ is the energy of collector; E_M_ for metal ion species; E_S_ for the binding model; E_B_ is the binding energy.

**Table 3:** The XRF analysis of wolframite.

Composition	WO_3_	MnO	Fe_2_O_3_	Al_2_O_3_	NiO	Ta_2_O_5_	CuO	TiO_2_	Others
Wolframite (%)	74.56	14.31	9.45	0.74	0.18	0.17	0.15	0.15	0.29

## Data Availability

Data are contained within the article.
